# 

**DOI:** 10.1192/bjb.2022.1

**Published:** 2023-04

**Authors:** Brendan D. Kelly

**Affiliations:** Professor of Psychiatry at Trinity College Dublin, Trinity Centre for Health Sciences, Tallaght University Hospital, Dublin, Ireland. Email: brendan.kelly@tcd.ie

**Figure:**
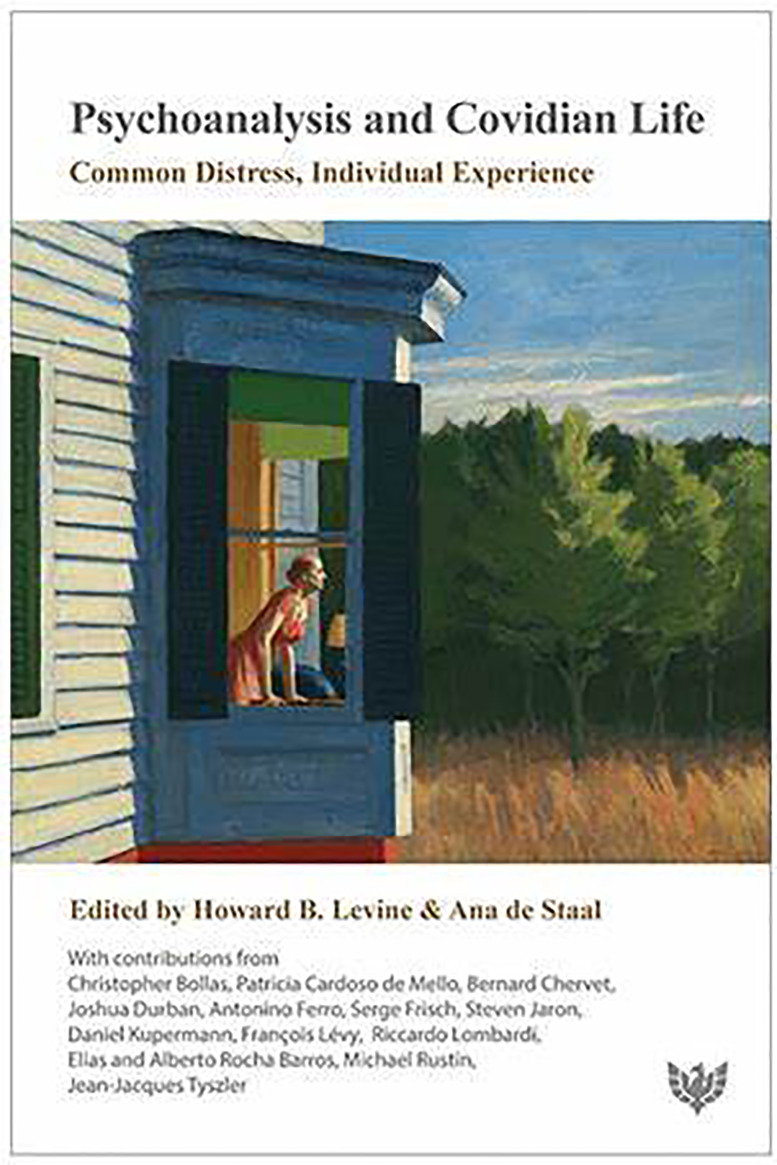

*Psychoanalysis and Covidian Life* focuses on the topic that has dominated so much of our lives and work in recent years: the COVID-19 pandemic. This interesting, thought-provoking volume opens with a quote from American writer H. P. Lovecraft: ‘The oldest and strongest emotion of mankind is fear, and the oldest and strongest kind of fear is fear of the unknown’.

How can we navigate the fear and unknowns of our current era? Can psychoanalysis help? If so, how?

This useful volume presents 15 interesting contributions that address this issue from various angles. It starts with an Editors’ note which highlights not only the impact of the pandemic on the structure of therapeutic sessions, but also its ‘potential to influence our practice more radically’.

I often struggle with psychoanalytic writing, but there is much to value in this accessible, engaging book. Michael Rustin starts his exploration of ‘the coronavirus pandemic and its meanings’ by going back to Trotsky's history of the Russian revolution. Rustin's idea of ‘progressive modernisation’ feels refreshingly immediate, relevant and direct in our current dilemma.

Ana de Staal writes about ‘psychoanalysis without a couch’, and Steven Jaron provides a searing account of ‘working in a post-ICU Covid-19 unit in a public hospital’ in Paris. Some of the clinical material in this chapter is very compelling and will be familiar to liaison psychiatrists. Jaron is acutely aware of his own journey through the quasi-apocalyptic landscape of a COVID-ridden city: ‘From a psycho-geographical perspective, the journey to the hospital aroused in my mind an image of a descent’.

Howard B. Levine concludes the book by noting that ‘the exigencies of the pandemic’ have given psychoanalysis an ‘unwelcomed and unexpected opportunity to rethink some very basic fundamental assumptions’. Of course, this is true not only of psychoanalysis, but of virtually every aspect of human life. The world has shifted in many respects.

The role of psychoanalysis in our new context will undoubtedly evolve further with time, but this book reaffirms that psychoanalysis indeed has a role, and it provides valuable notes on likely directions of future change.

